# Supplementary material for: Comprehensive Analysis of the Characteristics and Differences in Adult and Newborn Brown Adipose Tissue (BAT): Newborn BAT Is a More Active/Dynamic BAT
Source: Cells. 2020 Jan 14;9(1):201. doi: 10.3390/cells9010201 (PMC7017059; doi:10.3390/cells9010201)
Supplement: Supplementary file 1 [file cells-09-00201-s001.pdf]

Supplementary Materials:

Figure s. 1

A

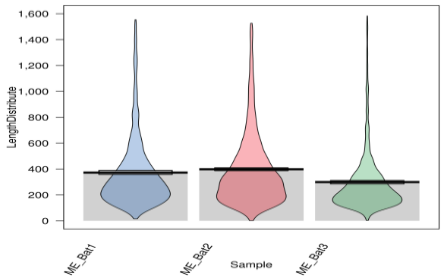

B

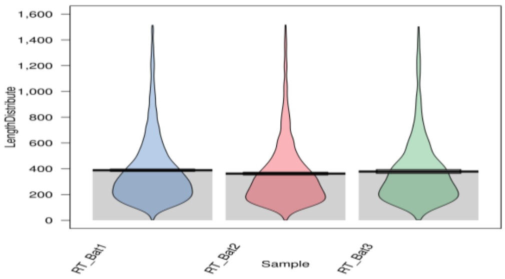

C

sample	Filter_circRNA	Candidata_circRNA
neBAT-1	7506	1323
neBAT-2	14677	2392
neBAT-3	12142	1403
aBAT-1	16139	3501
aBAT-2	12604	2510
aBAT-3	7916	1478

Figure s. 1. CircRNA analysis of aBATs and neBATs. (A and B) The length of most exonic circRNAs was < 1,500 nucleotides (nt), and the median length was about 400 nt. (C) Filtered circRNAs and candidate circRNAs in neBATs and aBATs.

Figure s. 2

A

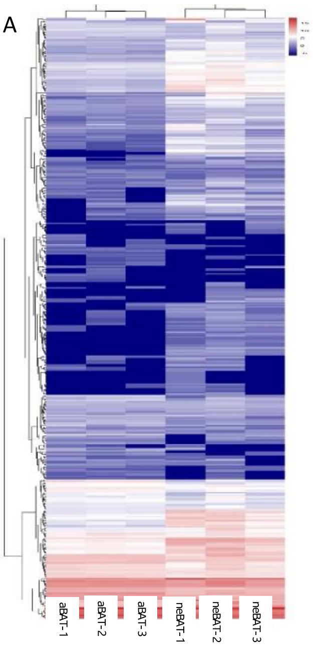

B

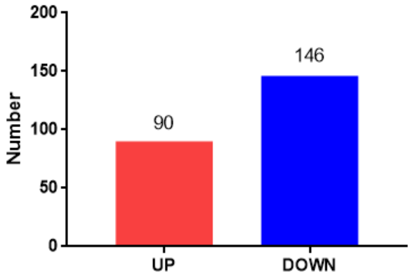

D

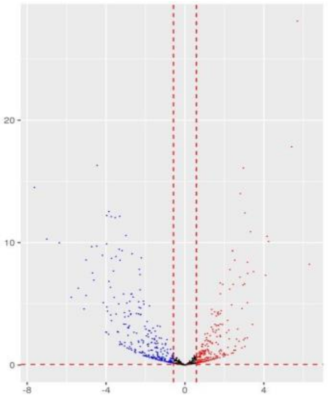

C

Sample	rRNA	tRNA	snoRNA	others	miRNA	New miRNA
ME_Bat_1	1,963,237	145,661	110,579	2,981,782	5,000,721	15
ME_Bat_2	65,374	443,510	57,905	1,401,551	4,523,520	8
ME_Bat_3	106,749	546,811	84,150	3,175,344	4,937,437	11
RT_Bat_1	520,501	643,184	66,906	2,061,754	6,846,961	13
RT_Bat_2	905,681	174,859	69,938	2,475,908	5,943,550	11
RT_Bat_3	2,122,347	186,349	104,270	3,801,419	6,297,062	14

Figure s. 2

E

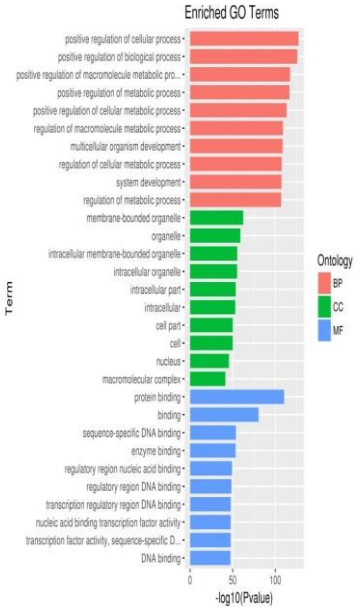

F

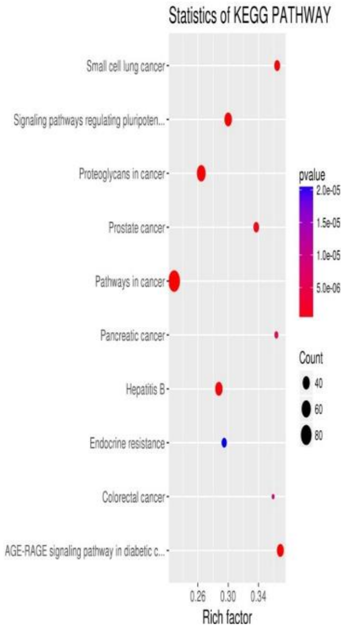

Figure s. 2. MicroRNA analysis of aBATs and neBATs (A) Heat map showing the differences in microRNAs between neBATs and aBATs. (B) Number of upregulated and downregulated microRNAs in neBATs and aBATs. (C) sRNA statistics in neBATs and aBATs. (D) Volcano plot of microRNAs. The volcano plots show significance on the y-axis (−log10, p-value) against the gene expression ratio (log2, fold change in neBATs vs. aBATs), and the FDR cutoff < 0.05 is indicated. (E) GO categories of differentially expressed microRNAs. (F) KEGG analysis of differentially expressed microRNAs. The number and p-value of differentially expressed microRNAs in each pathway.

Figure s. 3

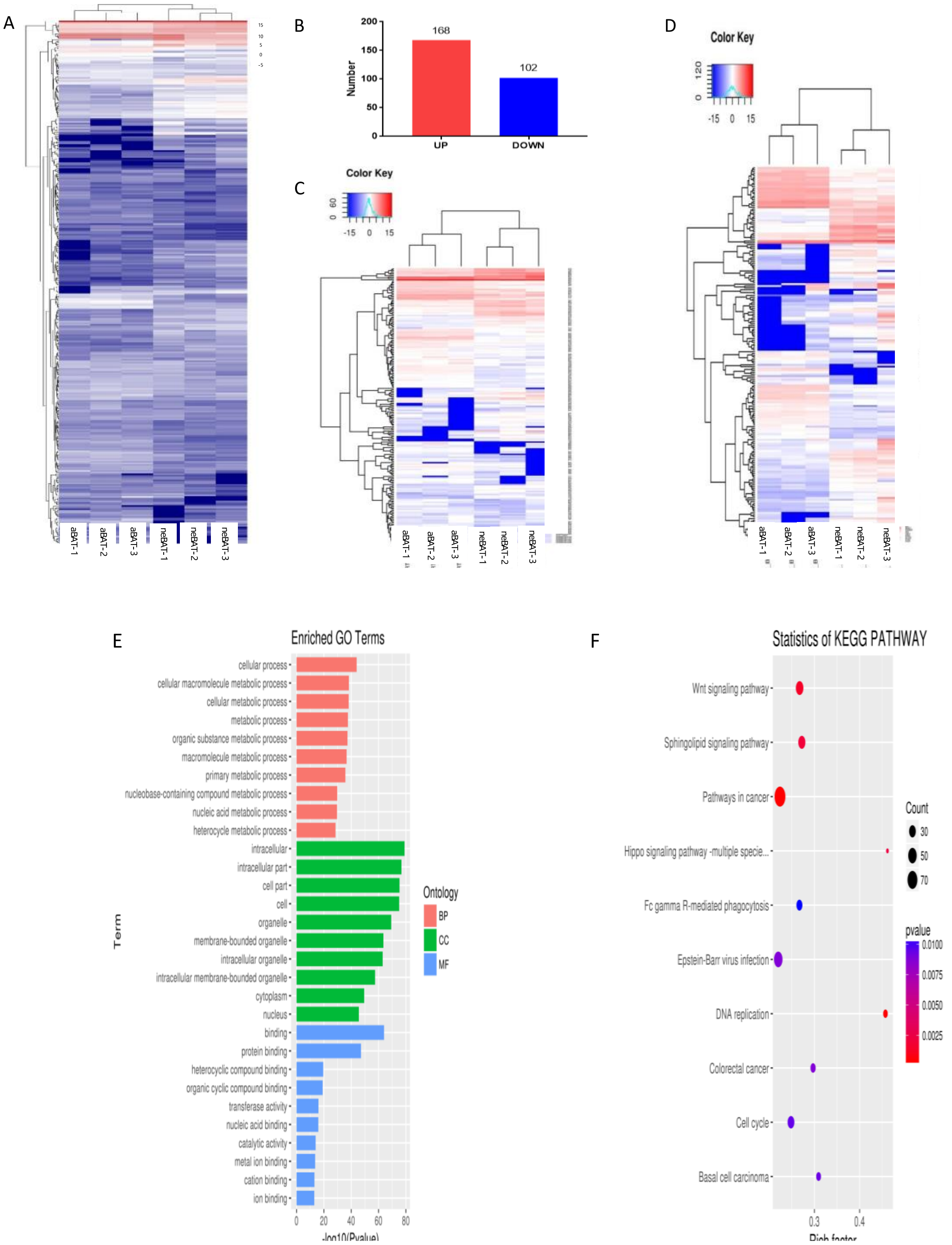

Figure s. 3. LncRNA analysis of aBATs and neBATs. (A) Heat map showing the differences in LncRNAs between neBATs and aBATs. (B) Number of upregulated and downregulated LncRNAs in neBATs and aBATs. (C) Heat map showing expression profiles of the top 200 differentially expressed lncRNAs. (D) Heat map showing expression profiles of the top 200 differentially expressed mRNAs. (E) GO categories for differentially expressed lncRNAs. (F) KEGG pathway analysis of differentially expressed lncRNAs. The number and p-value of differentially expressed lncRNAs.
